# A model assessment of the relationship between urban greening and ozone air quality in China: a study of three metropolitan regions

**DOI:** 10.1038/s41612-025-01054-4

**Published:** 2025-05-16

**Authors:** Jiawei Xu, Ben Silver, Rong Tang, Nan Wang, Xin Huang, Aijun Ding, Steve R. Arnold

**Affiliations:** 1 School of Atmospheric Sciences, Nanjing University, Nanjing, 210023 China; 2 Collaborative Innovation Center of Climate Change, Nanjing, Jiangsu Province 210023 China; 3 School of Earth and Environment, University of Leeds, Leeds, LS2 9JT UK; 4 Now at: Department of Earth and Environmental Sciences, University of Exeter, Penryn, TR10 9FE UK; 5 College of Carbon Neutrality Future Technology, Sichuan University, Chengdu, 610065 China

**Keywords:** Atmospheric chemistry, Atmospheric science

## Abstract

The impact of biogenic emissions on ozone (O_3_) has significant implications for air quality management. We analyze biogenic volatile organic compound (BVOC) emissions resulting from urban greening in three major Chinese cities, and impacts on tropospheric ozone. Urban greening BVOCs contributed 1.9 ppb (2.5%), 1.9 ppb (3.3%), and 3.6 ppb (5.9%) to O_3_ formation in Beijing, Shanghai, and Guangzhou, respectively. Temperature-driven enhancement in urban BVOCs produces significantly enhanced O_3_ on hot days. Guangzhou shows the highest summer temperatures, and the impact of the BVOC isoprene on O_3_ is more significant. The urban BVOC contribution to O_3_ is concentrated downwind of each city, due to transport processes. Estimated O_3_-related mortality in the cities was 900–2000 people during summertime, with 6–14% of the O_3_-related deaths attributable to urban BVOC emissions. The potential contribution of urban isoprene-emitting vegetation to air quality should be considered alongside the potential benefits of urban greening in future policy-making decisions.

## Introduction

Surface ozone (O_3_) is an important atmospheric pollutant that poses a risk to both human health and vegetation^1,2^. Tropospheric O_3_ is a secondary pollutant formed through the photochemical oxidation of volatile organic compounds (VOCs) in the presence of nitrogen oxides (NO_X_)^3,4^. In recent years, O_3_ pollution has become a major concern in China^5–7^. From 2013 to 2017, the summer daily maximum 8-h averages of O_3_ concentration (MDA8 O_3_) increased by 3–12 ppb per year in Chinese megacities^7^. Despite numerous efforts to implement emission reduction policies to control O_3_ pollution^8–10^, the complex relationship between O_3_ and its emitted precursors presents significant challenges.

O_3_-vegetation interactions, ranging from regional to global scales, are an important consideration in understanding the formation and environmental impacts of O_3_ pollution^11–13^. Vegetation is the largest contributor to VOCs worldwide^14^. In China, it is believed that emissions of biogenic VOCs (BVOCs) are as large as those from human activities^15,16^. BVOCs are highly reactive, providing an efficient source of peroxy radicals to drive O_3_ formation, and are precursors for organic nitrates and peroxyacetyl nitrate (PAN)^17,18^. Extensive research highlights the importance of BVOCs for O_3_ pollution, and the contribution of BVOCs to O_3_ may be comparable to that of anthropogenic VOCs (AVOCs) in some cases^19,20^. Therefore, fully exploring the effects of BVOCs on O_3_ in China is essential for accurately understanding the formation of O_3_ pollution and for fully understanding the efficacy of policies aimed at reducing O_3_ pollution through controls on anthropogenic VOC emissions.

O_3_ pollution in China is known to be sensitive to emissions of BVOCs. In the Beijing–Tianjin–Hebei (BTH) region of northern China, BVOCs have been found to contribute more than 10% to surface O_3_ during an extreme heat event^15^. Liu et al.^19^ analyzed a case of O_3_ pollution in the Yangtze River Delta (YRD) region and found that BVOCs contributed up to 27 ppb to O_3_, accounting for 11% of the average monthly O_3_ formation in the region. In the Pearl River Delta (PRD) region, the average contribution of summertime BVOCs to O_3_ is 10 ppb, with a maximum of 34 ppb^16^. Additionally, the oxidation products of BVOCs can be transported to downwind regions, causing high O_3_ concentrations^21^. During a typhoon event, the typhoon-boosted biogenic emissions and regional transport of O_3_ and precursors resulted in elevated O_3_ in both the YRD and PRD regions^22^. However, previous studies may have underestimated both the emissions of BVOCs from urban areas and their impacts on O_3,_ due to the coarse resolution of the models used.

The ongoing urbanization process in China has seen a marked focus on urban greening^23,24^. This expansion of urban green spaces has led to a consequent increase in biogenic emissions in urban areas^15,25^. Some researchers have noted that many previous studies have widely underestimated or ignored the BVOC emissions from urban areas^15,26,27^. For example, greening in Beijing has doubled the city-wide total BVOC emissions between 2005 and 2010^28^. A study by Ren, Qu^29^ found that emissions from the urban landscapes accounted for 15% of total BVOC emissions in Beijing in 2015. Despite lower vegetation densities in urban areas, urban BVOC emissions may still contribute significantly to O_3_, since the large localized NOx emissions in urban areas mean that they are typically in a VOC-limited regime, in which an increase in VOC emissions leads to an increase in O_3_^29^. Moreover, urban vegetated landscapes may have greater relative emissions of BVOCs than natural forests due to favorable conditions such as lower tree densities and better light exposure^25^. Therefore, further exploration is needed to gain a deeper understanding of the relationship between urban vegetation sources of BVOCs and O_3_ air quality.

Here, we utilize a widely used land-use dataset from the Moderate Resolution Imaging Spectroradiometer (MODIS) and a high-resolution land-use dataset (FORM-GLC10) to assess biogenic emissions in three urban areas of China. A coupled chemical-transport model (WRF-CMAQ) is used to evaluate the impacts of urban greening BVOCs on O_3_ air quality in these regions. Our focus is on three major city clusters in China: BTH, YRD, and PRD, with the center city of each cluster, Beijing, Shanghai, and Guangzhou, respectively, being selected for analysis. Finally, we use these results to calculate the health impacts of increased surface O_3_ resulting from urban BVOC emissions in the representative cities.

## Results

### Urban biogenic emissions in three major cities

**Fig. 1:**
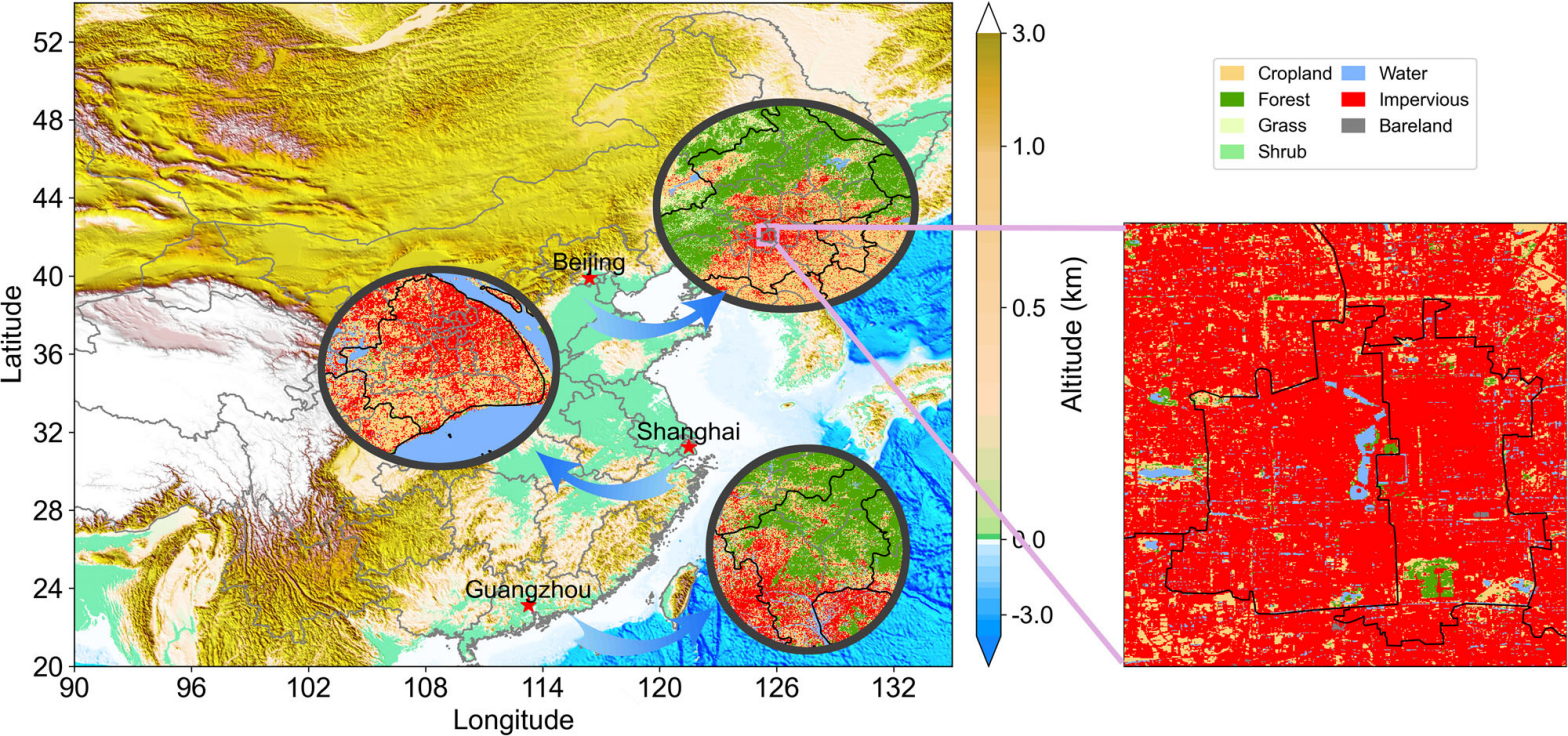
Locations of Beijing, Shanghai, and Guangzhou, within a topographic map of China. Three circle panels show the land cover categories within the three cities at a resolution of 10 m. The right rectangle panel shows the 10 m land use of the central urban area of Beijing.

In this study, we focus on quantifying impacts of urban greening BVOC emissions in the urban centers of China’s three major city clusters, namely Beijing (the capital and the central city of the BTH region), Shanghai (the central city of the YRD region) and Guangzhou (the central city of the PRD region). All three cities have undergone rapid urbanization in recent decades, resulting in large increases in anthropogenic emissions^8,30,31^. At the same time, due to social and environmental benefits and to help the health of urban residents, municipal governments have made efforts to protect and construct green space^32,33^. Figure 1 shows the topography and land use types of the three cities. They are all located in low-altitude areas without significant terrain variation. The 10 m resolution land use datasets have allowed us to accurately map the distribution of vegetation within urban areas, revealing previously unresolved urban green spaces such as Tiantan Park in Beijing (the green area shown in the bottom right corner of the right panel, covering an area of about 273 ha). In contrast, traditional MODIS satellite datasets, with their coarser resolution (2500 ha per unit grid), cannot accurately represent such urban green spaces. As a result, the MODIS landuse type in the center of Beijing in Fig. 1 is classified as urban type without vegetation. FORM-GLC10 effectively solves the problem of resolution and enables the calculation of emissions from urban greening at fine resolution.

**Fig. 2:**
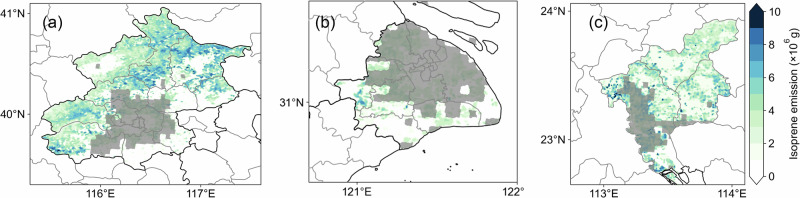
Simulated total summertime (JJA) regional isoprene emissions. Gridded emissions are plotted for **a** Beijing, **b** Shanghai, and **c** Guangzhou. Urban areas identified in MODIS data are indicated by gray shading.

In this study, we simulated isoprene (C_5_H_8_, the major component of BVOCs) emissions in the three cities using 10 m high-resolution land use (Fig. 2a–c). The outer suburbs of the cities usually have the highest biogenic emissions, with specific sources including Yanshan Mountain to the north of Beijing, Dianshan Lake to the west of Shanghai, and forests in the Conghua district to the north of Guangzhou. The urban areas (gray areas) are identified using MODIS data, while the whole city is identified based on administrative boundaries. The total city-wide annual summertime emissions (urban areas) of Beijing, Shanghai, and Guangzhou are 46.09 Gg (7.39 Gg), 8.67 Gg (6.01 Gg), and 23.66 Gg (6.40 Gg), respectively. Our simulated isoprene emissions are consistent in magnitude with previous work^34,35^.

### O_3_ enhancement due to urban greening BVOCs

**Fig. 3:**
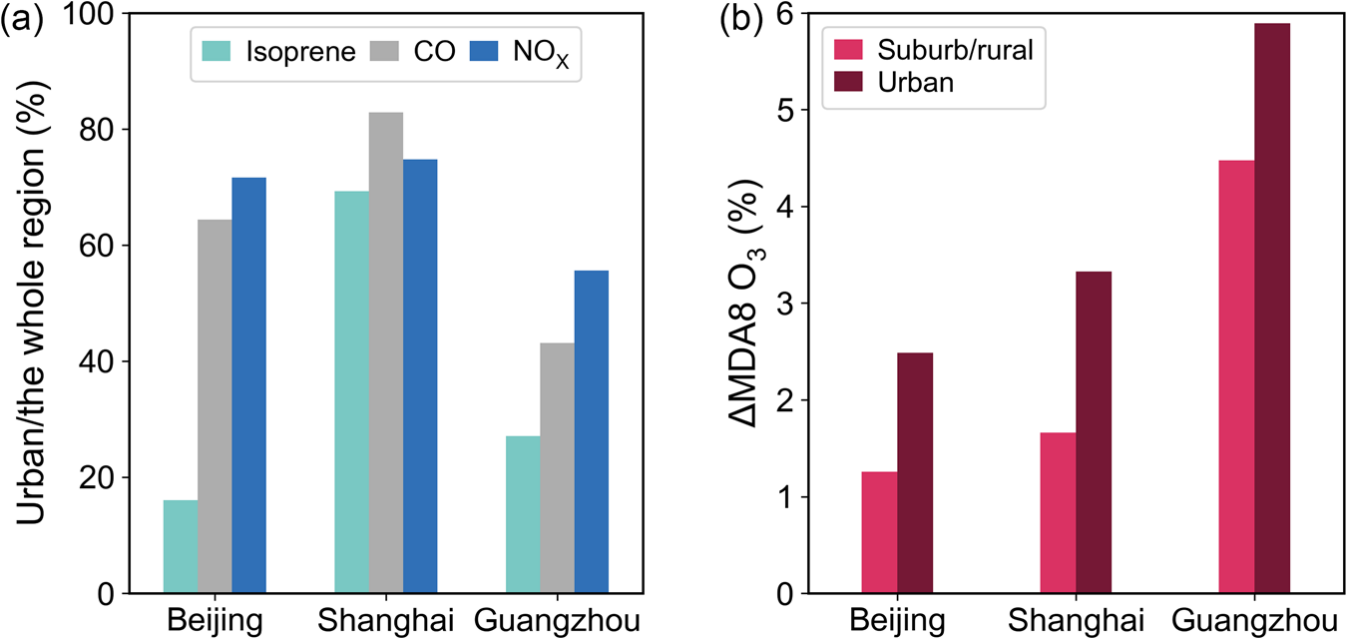
Comparison of urban and non-urban emissions and ozone air quality responses across the three study regions. **a** Ratio of emissions of isoprene, CO, and NO_*x*_ in urban areas to the entire city region. **b** Relative changes of MDA8 O_3_ due to urban BVOCs in both urban and suburban/rural areas.

Simulations of biogenic emissions in urban areas allow us to evaluate the contribution of urban greening to biogenic emissions and O_3_ air quality. The formation of O_3_ is affected by both anthropogenic and biogenic sources, which further determine O_3_ sensitivity regimes^36^. Anthropogenic emissions are concentrated in urban areas in all three cities, with NO_X_ emissions in urban areas accounting for more than 70% of total NO_X_ emissions in Beijing and Shanghai, and approximately 60% in Guangzhou (Fig. 3a). In contrast, emissions from vegetation are mainly concentrated in the suburbs. The ratios of isoprene emissions in the urban areas to the whole metropolitan areas are only around 15–25% in Beijing and Guangzhou. Particularly, isoprene emissions from Beijing urban areas account for around 15% of total isoprene emissions, consistent with a previous vegetation survey^25^. Shanghai has a higher ratio due to its classification of most grids as urban type. Since the urban areas are usually determined as VOC-limited regimes, O_3_ concentrations increase with increasing BVOC emissions.

In all cities, the urban-BVOC-emission-induced O_3_ enhancement is stronger in urban areas than in suburban areas (Fig. 3b). The average summer contribution of urban greening BVOCs to MDA8 O_3_ is 1.9 ppb (2.5%) in Beijing, 1.9 ppb (3.3%) in Shanghai, and 3.6 ppb (5.9%) in Guangzhou. Among the three cities, Guangzhou shows the greatest impact due to higher vegetation density and higher average summer temperatures compared with Beijing and Shanghai^37^.

**Fig. 4:**
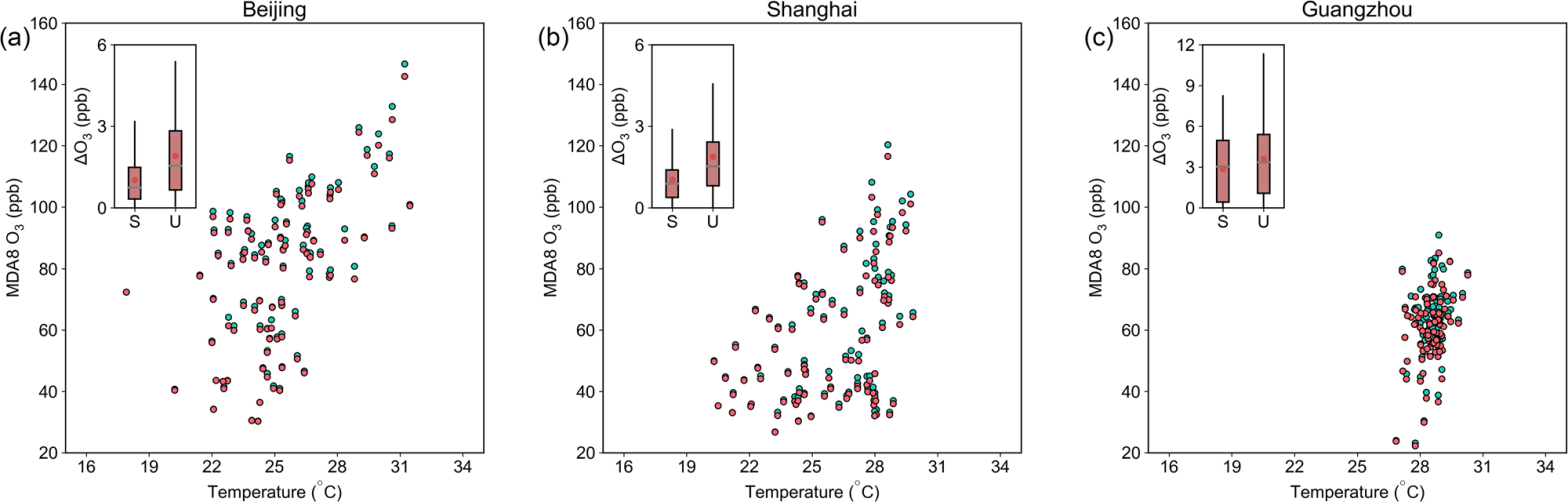
Relationships between 2 m temperature and MDA8 O_3_ for urban greening and base scenarios. Relationships are plotted for **a** Beijing, **b** Shanghai, and **c** Guangzhou. The urban greening scenario is labeled as “UG scenario” (in cyan), while the scenario without urban vegetation emissions is the “Base scenario” (in red). Impacts of BVOCs from urban greening on O_3_ in urban and suburban/rural areas are shown as boxplots. Note that the vertical lines represent the minimum and maximum values, the boxes mark the 25th, 75th, and 50th percentiles, the horizontal line represents the mean value, and the dots inside the box indicate the mean. The labels “S” and “U” indicate suburban/rural and urban areas, respectively.

We analyze the relationship between surface temperature and O_3_ in all three cities under the two model scenarios. O_3_ concentrations tend to increase as temperature increases (Fig. 4) due to enhanced biogenic emissions, accelerated PAN decomposition, or lower humidity^38–40^. In our simulations, we found that there is a positive correlation between both O_3_ concentrations and the contribution of urban greening to O_3_ with temperature. We investigated the relationship between the difference in O_3_ produced by urban greening BVOCs (UG scenario minus Base scenario) and surface temperature in the three cities (Fig. S2). Correlation coefficients are significant in Beijing (*r* = 0.49, *p* = 9.5 × 10^−^^7^) and Shanghai (*r* = 0.57, *p* = 3.3 × 10^−9^), with no significant correlation found in Guangzhou. When the average daily temperature reaches 30 °C, the O_3_ contribution from urban greening BVOCs in Beijing and Shanghai reaches its maximum (Fig. 4a, b). While Guangzhou experiences a similar pattern, the frequent occurrence of extreme heat days in the city makes the impact of urban greening much greater compared to the other two cities (Fig. 4c). Therefore, we may expect the impacts of urban greening on O_3_ via BVOC emissions to be larger under increasing surface temperatures^41^.

Furthermore, our study shows that the O_3_ enhancement from urban greening BVOC emissions in Beijing and Shanghai can reach up to 4 ppb, while in Guangzhou, the enhancement can exceed 10 ppb. The isoprene increases caused by urban greening in all three cities are around 1 ppb on extreme heat days (Fig. S3). The differences in concentration changes of isoprene between urban and suburban areas are substantial. For example, when accounting for urban greening, isoprene concentrations increase by approximately 500% in urban areas, while in suburban areas, the increase is only about 5%. Isoprene is highly reactive and quickly transforms into other reactants^3,42,43^, so its concentration in the suburban areas may change only slightly even when isoprene emissions in the urban areas change significantly.

**Fig. 5:**
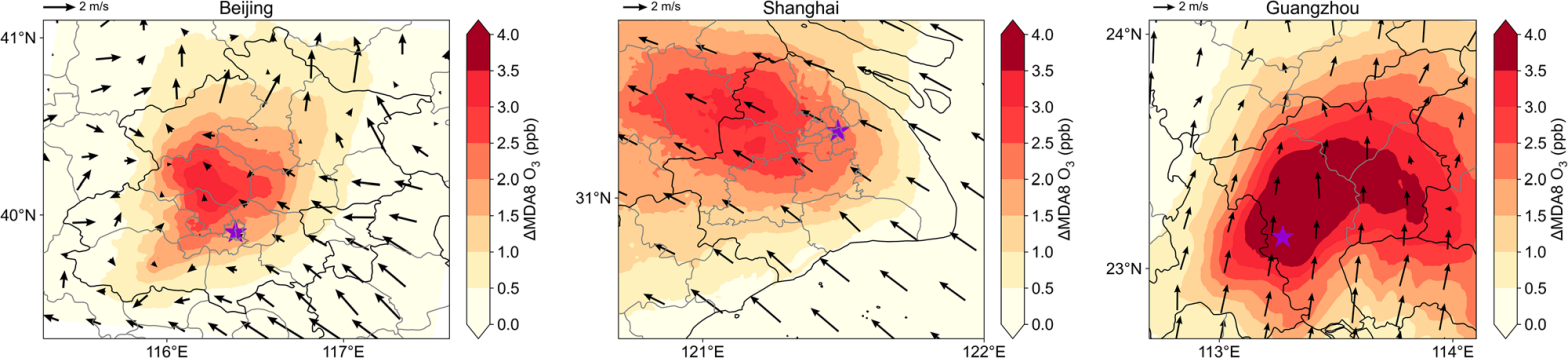
Regional distributions of 10 m wind and impacts of urban greening on O_3_. Panels show the three study regions of interest, and purple stars represent each respective city center location.

The regional transport of BVOCs has been discussed in previous studies^22,44^. While BVOCs emitted from forests have short lifetimes (~20 min for isoprene^43^), their products have longer lifetimes and can be transported. In our study, spatial analysis reveals that the impact of urban greening BVOCs on air quality is not limited to local areas but also extends to areas downwind of each of the city areas (Fig. 5). However, since the total amount of BVOCs emitted by urban green spaces is still much less than those from forests, the affected areas are not as large. We recommend that policymakers consider upwind and downwind influences when planning residential areas to avoid impacts on populations in downwind areas. This approach can help safeguard the health of residents from unexpected effects of increases in BVOC emissions upwind.

### Evaluation of health impacts

**Fig. 6:**
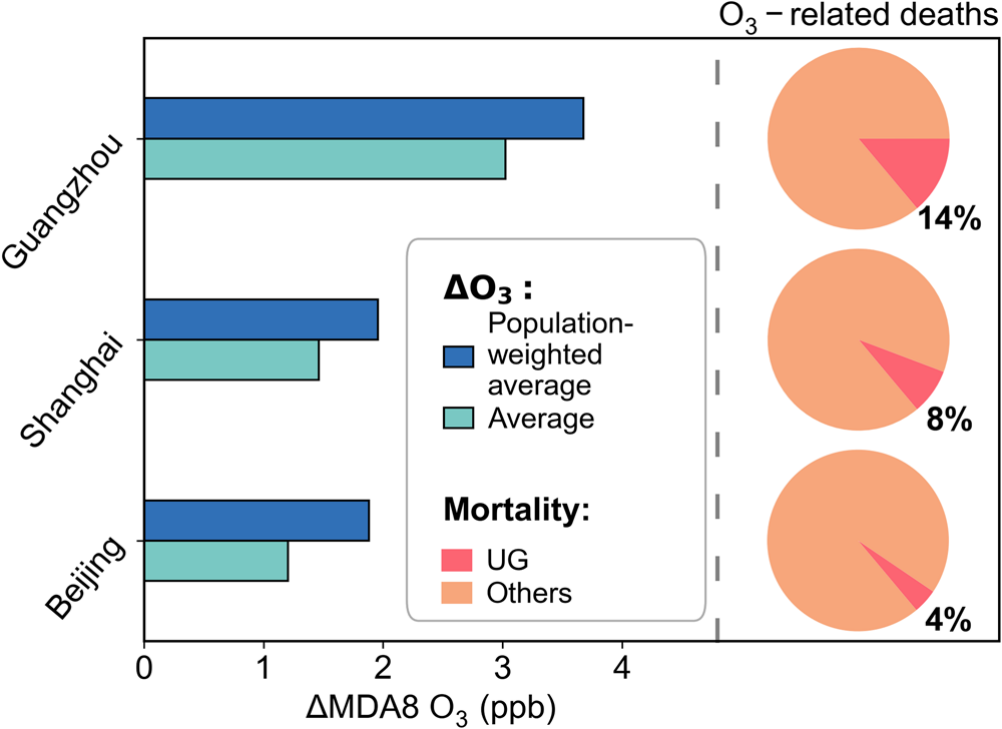
Comparison of O_3_ changes and health effects from urban greening BVOC emissions in three cities. Left bars represent the population-weighted average and numerical average of O_3_ changes. The right pie charts represent O_3_-related deaths. The percentages in the bottom right corners of the pie charts show the fraction of deaths related to ozone increases caused by urban greening BVOCs.

As urban areas are densely populated, it is important to evaluate the health effects of changes in urban O_3_ induced by urban greening. We find that the increase of population-weighted average O_3_ caused by urban greening BVOCs is higher than the simple spatial average, indicating that O_3_ is more affected in populated urban areas (Fig. 6). Based on the method summarized in Section “Evaluation of health impacts”, we calculate O_3_-related premature mortality in all three cities. In the summer of 2019, the number of deaths due to O_3_ exposure was 1182, 758, and 458 in Beijing, Shanghai, and Guangzhou, respectively. Among these deaths, those resulting from urban greening BVOCs accounted for 4–8% of total ozone-related deaths in Beijing and Shanghai, and 14% in Guangzhou. These findings suggest that Guangzhou should be particularly mindful of tree species (and propensity for isoprene emissions) and population distribution in urban planning to mitigate the negative effects of urban greening BVOCs via O_3_ pollution.

## Discussion

Understanding the mechanisms that drive O_3_ pollution and its distribution is a crucial topic in China, particularly in light of the country’s aspirations for “blue sky” and “carbon neutrality” in the future^45,46^. As anthropogenic emission reduction policies are implemented, and as temperatures increase with climate warming, the impacts of vegetation become an increasingly important factor driving reactive VOC emissions^47^. Our study focused on the impacts of BVOCs from urban green spaces on O_3_ in the central cities of the three major urban clusters, namely Beijing, Shanghai, and Guangzhou. Our findings suggest that although biogenic emissions in urban areas are significantly lower than those in suburban areas, urban greening impacts on BVOC emissions can still contribute to 1.9 ppb (2.5%), 1.9 ppb (3.3%), and 3.6 ppb (5.9%) of O_3_ formation in these three cities. We also analyzed the impact of urban greening biogenic emissions on O_3_ and isoprene under different temperature conditions. Our results show that on extreme heat days, the daily MDA8 O_3_ in urban areas is significantly higher than the average level, and the isoprene concentration is well correlated with the temperature. For example, the simulated O_3_ in Shanghai captured the pattern of observational O_3_ on 12 August when the BVOC emissions from urban greening are considered (Fig. S4). In this case, Shanghai was located in the saddle-shaped field formed by two typhoons (“Lekima” and “Krosa”) as shown in Fig. S5, leading to stable weather conditions. The BASE experiment failed to simulate the magnitude of observed O_3_ concentrations. After including the emissions and effects of BVOCs from urban greening (UG scenario), the simulated daytime O_3_ concentration increased by more than 20 ppb compared with the BASE experiment. These results suggest that urban vegetation BVOC emissions are important in driving elevated O_3_ concentrations during extreme heat events. It is worth noting that the contribution of urban greening BVOCs to O_3_ is concentrated downwind areas of each city, emphasizing the importance of transport processes in shaping air quality. Furthermore, our analysis indicates that O_3_ exposure leads to 900–2000 premature mortalities in these cities, with the impact of urban greening BVOCs particularly significant in Guangzhou, where it accounts for 14% of summertime O_3_-related deaths. In the future, the impact of BVOCs on secondary organic aerosols (SOA) in urban regions should also be explored. Liu et al.^48^ found that BVOCs in China would increase by 11.13% in 2050 in the RCP4.5 scenario, leading to an increase of SOA by 6.5%. Our study found that urban vegetation added an additional 15–25% to BVOC emissions in major cities. These discoveries suggest the impact of SOA deserves further consideration in future research.

We have isolated the impact of BVOC emissions from urban vegetation on O_3_ air quality in three key city regions of China. However, quantifying the overall net impacts of urban green spaces on population health is more complex, and should include an assessment of air quality impacts via changes in dry deposition^49,50^, modifications to local surface heat and moisture fluxes^51–53^, and urban temperatures^54,55^, as well as potential mental health and wellbeing benefits of increased green space access^56–58^. Additionally, it is worth mentioning that urban greening also has many benefits, such as reducing flood risk^59^. Nevertheless, our results contribute an important constraint on local and regional-scale air quality impacts of urban-scale biogenic emissions, and are useful for informing decision-making around enhancing tree cover in populated city regions. As a priority, we recommend that emissions of AVOCs should be reduced. By AVOC emissions control, cities can effectively address air quality concerns despite potential increases in BVOCs and promote a healthier urban environment.

## Methods

### Observational and reanalysis data

Ground-based observations of 2019 summertime (JJA) O_3_, nitrogen dioxide (NO_2_), CO, particulate matter 2.5 (PM_2.5_) concentrations at more than 1400 stations are used to evaluate our model simulations. Hourly monitoring data are archived at the air monitoring data center of the Ministry of Ecology and Environment (MEE) of China. In 2013, MEE started to establish monitoring sites in major cities, which were later expanded to cover most cities in China. Here, we evaluate model-simulated concentrations using these observations within megacity clusters.

### Regional chemical transport model

The Weather Research and Forecast—Community Multiscale Air Quality (WRF-CMAQ) modeling system is employed to investigate the effects of urban greening on atmospheric composition in China. This modeling system considers complex meteorology–chemistry interactions and has been widely used to understand the impacts of meteorology and emission changes on air pollution^8,60,61^.

The modeling system consists of two parts. The dynamical model, WRFv3.8.1, is a mesoscale numerical weather prediction system designed for operational forecasting and atmospheric research. In this study, three domains are designed with horizontal resolutions of 25 km, 5 km, and 1 km (Fig. S1). The first domain (25 km resolution) covers the whole of China and its surrounding areas, centered at 39°N, 106.8°E. There is a total of 29 vertical layers with top pressure at 100 hPa. One-way nested runs are conducted from 25 to 1 km. Atmospheric chemistry is simulated using CMAQv5.1. The second model component considers gas phase chemistry, represented by Carbon Bond version 05 (CB05) combined with Aerosol Module version 6 (AERO6). The boundary conditions of the first domain are created by the Community Earth System Model (CESM) from previous work^5^. The key configuration of WRF-CMAQ includes the Rapid Radiative Transfer Model (RRTM) for longwave and shortwave radiation, the Noah Land Surface Model for land-atmospheric interactions, the Kain-Fritsch scheme for cumulus parameterization, the Lin microphysics scheme, and the ACM2 boundary layer scheme. The anthropogenic emissions of China are obtained from the Multi-resolution Emission Inventory for China (MEIC), developed by Tsinghua University^62^. The emission inventory is from 2017, available with two horizontal resolutions, 0.25° and 0.1°. Here, emissions with a 0.25° × 0.25° grid are regridded to the two coarser model grids (25 km and 5 km). For the third domain (1 km), we remapped the 0.1° higher resolution anthropogenic emissions (BC, CO, SO_2_, NO_*X*_, PM_2.5_, PM_10_) in our simulation. Note that for emissions of VOCs we retain the 0.25° × 0.25°, since MEIC does not include 0.1° × 0.1° resolution data for condensed VOCs of the CB05 mechanism.

The biogenic emissions in our simulations are calculated online using the Model of Emissions of Gases and Aerosols from Nature version 2.1 (MEGANv2.1)^63^. MEGAN is widely used in simulations of BVOCs^16,19,37,64^. MEGANv2.1 calculates emissions of more than 100 biogenic VOCs, using the function:

1 $${\rm{F}}_{\rm{i}}={\gamma }_{\rm{i}}\sum {{\rm{\epsilon }}}_{\rm{i},{\rm{j}}}{{\chi }}_{\rm{j}}$$

where *F*_*i*_, *ε*_*i,j*_, and *χ*_*j*_ are emission amount, standard emission factor, and fractional coverage of each plant functional type (PFT) *j* of chemical species *i*. *γ*_*i*_ is the emission activity factor, which is calculated based on canopy environment coefficient (C_CE_), leaf area index (LAI), light (*γ*_*L*_), temperature (*γ*_*T*_), leaf age (γ_LAI_), soil moisture (γ_SM_), and CO_2_ inhibition (γ_CI_):

2 $${\gamma }_{i}={C}_{{CE}}{{LAI}}{\gamma }_{L,i}{\gamma }_{T,i}{\gamma }_{{{LA}},i}{\gamma }_{{{SM}},i}{\gamma }_{{{CI}},i}$$

In the two coarser domains, PFT data is taken from MODIS MCD12Q1 datasets and is classified from 8 vegetation types, transposed to the 16 PFT types in MEGANv2.1 according to Bonan et al.^65^. LAI data is from the MODIS MCD15A2H dataset. LAI products are composited every 8 days^66,67^. However, these datasets do not include greening grid squares in urban areas because of their coarse resolution. In the third 1 km resolution domain, we retain the use of MODIS datasets in suburban and rural areas. However, in urban areas, an alternative land cover dataset, FROM-GLC10, is used^68^. FROM-GLC10 has a higher spatial resolution of 10 m, clearly resolving the distribution of street trees, parks and other greenspaces in urban areas. 4 vegetation types of FROM-GLC10 are classified into 16 PFT types according to Ma, Gao^35^. In this analysis, all tree cover grid cells in urban areas are assumed to be broadleaf trees. The classification of a broadleaf tree as either evergreen or deciduous is based on its latitude. The finer resolution grid LAI is calculated based on an empirical relationship between PFTs and LAI^69^.

### Estimation of the health impacts

Ambient air pollution, including exposure to ground-level O_3_, is a major global health concern^70^. O_3_ has been shown to have significant impacts on human health, leading to or exacerbating cardiopulmonary and respiratory diseases^71–74^. We estimate the health effects of exposure to ambient O_3_ based on our model-simulated O_3_ distributions in the three urban regions considered. Based on the calculation approach for health impact estimation adjusted by Apte et al.^75^, we evaluated O_3_-attributable deaths associated with chronic obstructive pulmonary disease (COPD). The attributable-fraction type relationship presented in (3) was used to calculate the mortality attributable to outdoor O_3_ exposure:

3 $${\rm{M}}_{{\rm{i}},{\rm{j}}}=\sum _{\rm{g}}{\rm{P}}_{{\rm{g}},{\rm{j}}}\times {\hat{\rm{I}}}_{{\rm{i}},{\rm{j}}}\times ({\text{RR}}_{{\rm{g}},{\rm{i}},{\rm{j}}}\left({\rm{C}}_{\rm{g}}\right)-1)$$

4 $${\hat{\rm{I}}}_{{\rm{i}},{\rm{j}}}={\rm{I}}_{{\rm{i}},{\rm{j}}}/\overline{{\text{RR}}_{{\rm{g}},{\rm{i}},{\rm{j}}}}$$

5 $$\overline{{\text{RR}}_{{\rm{g}},{\rm{i}},{\rm{j}}}}=\frac{\mathop{\sum }\nolimits_{{\rm{i}}=1}^{\rm{N}}{\rm{P}}_{{\rm{g}},{\rm{j}}}\times {\text{RR}}_{{\rm{g}},{\rm{i}},{\rm{j}}}\left({\rm{C}}_{\text{g}}\right)}{\mathop{\sum }\nolimits_{{\rm{i}}=1}^{\rm{N}}{\rm{P}}_{{\rm{g}},{\rm{j}}}}$$

where *M*_*g,i,j*_ is the mortality of disease *i* and age group *j* in grid cell *g*; *P*_*g,i*_ is the population; *I*_*i,j*_ is the reported national average annual disease (mortality) incidence rate; *C*_*g*_ represents the O_3_ concentration; RR_*g,i,j*_(*C*_*g*_) is the relative risk at concentration *C*_*g*_; $\overline{{\text{RR}}_{{g},{i},{j}}}$ represents the average population-weighted relative risk; ${\hat{{I}}}_{{i},{j}}$ is the hypothetical “underlying incidence” (cause-specific mortality rate) that would remain if O_3_ concentrations were reduced to the theoretical minimum risk concentration (i.e., 32.4 ppb^76^).

6 $${\text{RR}}_{{g},\text{i},{j}}\left({\rm{C}}_{{g}}\right)={{rr}}_{g,i,j}^{({C}_{g}-{C}_{0})/10}\,{{{\rm{if}}\;{\rm{ C}}}}_{{g}} \,>\, 32.4$$

where *rr* is the increased risk of mortality associated with a 10 ppb increase according to a previous study.

Here, we apply the disease incidence in 2015 derived from the Global Burden of Disease Results Tool 2017 version (GBD2017)^77^. The population size and spatial distribution for 2015 at 0.0083° × 0.0083° (30”) resolution are obtained from the Gridded Population of the World (GPW)^78^. Age structure at the national level in 2015 is also from GBD2017.

### Experimental design

Two simulation scenarios are performed to investigate the effects of BVOC emissions from urban greening on regional O_3_ concentrations. The simulations are conducted from 28th May to 31st August 2019. For all simulations, the first 4 days are considered model spin-up. The Base scenario is set to exclude all the BVOC emissions in urban areas. A second simulation that includes all BVOC emissions (named as UG) allows quantification of the impacts of urban greening BVOC emissions compared with the results of the base scenario. The results of the urban greening scenario demonstrate that the model captures O_3_ changes in summer in three representative cities, giving us confidence in our subsequent analysis (Tables S1 and S2).

## Supplementary information

Supplementary Tables

## Data Availability

Data that support the findings of this study are available from the authors on request.

## Code availability

The model source code used in this study is publicly available for the CMAQ model at https://github.com/USEPA/CMAQ and for WRF at https://github.com/wrf-model/WRF. The MEGAN source code, which is used to calculate biogenic emissions, is available at https://bai.ess.uci.edu/megan/data-and-code/megan21. Python scripts used in the analysis and production of figures are available from the corresponding author on reasonable request.
