# Supplementary material for: A model assessment of the relationship between urban greening and ozone air quality in China: a study of three metropolitan regions
Source: NPJ Clim Atmos Sci. 2025 May 16;8(1):184. doi: 10.1038/s41612-025-01054-4 (PMC12084151; doi:10.1038/s41612-025-01054-4)
Supplement: Supplementary file 1 — Supplementary Tables [file 41612_2025_1054_MOESM1_ESM.pdf]

A model assessment of the relationship between urban greening and ozone air quality in China: a study of three metropolitan regions

Jiawei Xu[1,2,3], Ben Silver[3], Rong Tang[1,2], Nan Wang[4], Xin Huang[1,2], Aijun Ding[1,2], Steve R. Arnold[3,*]

Key words: High resolution, Urban greening, Ozone (O_3), urban pollution, health impacts

1 School of Atmospheric Sciences, Nanjing University, Nanjing, 210023, China

2 Collaborative Innovation Center of Climate Change, Jiangsu Province, Nanjing, 210023, China

3 School of Earth and Environment, University of Leeds, Leeds, LS2 9JT, UK

4 College of Carbon Neutrality Future Technology, Sichuan University, Chengdu, 610065, China

Table S1. Evaluation of meteorological results in Beijing

	Parameter	Obs_{mean}	Sim_{mean}	MB	NMB	NME	RMSE	IOA
D01	T (°C)	26.65	25.51	-1.13	-0.04	0.07	2.38	0.92
	RH (%)	58.55	56.93	-1.12	-0.02	0.14	10.55	0.92
	P (hPa)	1000.95	983.81	-17.24	-0.02	0.02	17.27	0.75
	U (m/s)	-0.16	-0.02	0.11	-0.69	-7.46	1.49	0.71
	V (m/s)	0.19	0.31	0.18	0.9	6.95	1.69	0.82
D02	T (°C)	26.65	28.08	1.42	0.05	0.08	2.76	0.91
	RH (%)	58.55	49.43	-8.63	-0.15	0.21	15.24	0.86
	P (hPa)	1000.95	998.12	-2.93	0	0	3.29	0.87
	U (m/s)	-0.16	-0.55	-0.37	2.35	-11.99	2.43	0.52
	V (m/s)	0.19	0.64	0.49	2.51	10.19	2.53	0.71
D03	T (°C)	26.65	27.53	0.88	0.03	0.08	2.74	0.9
	RH (%)	58.55	53.25	-5.07	-0.09	0.19	14.77	0.87
	P (hPa)	1000.95	997.68	-3.36	0	0	3.73	0.85
	U (m/s)	-0.16	-0.52	-0.35	2.22	-12.98	2.64	0.49
	V (m/s)	0.19	0.78	0.61	3.14	12.3	3.12	0.63

Table S2. Evaluation of O_3 results in three cities

	Obs_{mean}	Sim_{mean}	MB	NMB	NME	IOA
Beijing						
d03_UG	51.91	44.54	-7.36	-0.14	0.45	0.79
d03_BASE	51.91	43.47	-8.44	-0.16	0.45	0.79
Shanghai						
d03_UG	41.77	30.48	-11.29	-0.27	0.52	0.74
d03_BASE	41.77	29.73	-12.04	-0.29	0.52	0.73
Guangzhou						
d03_UG	27.58	26.03	-1.55	-0.04	0.58	0.82
d03_BASE	27.58	24.44	-3.14	-0.1	0.56	0.82

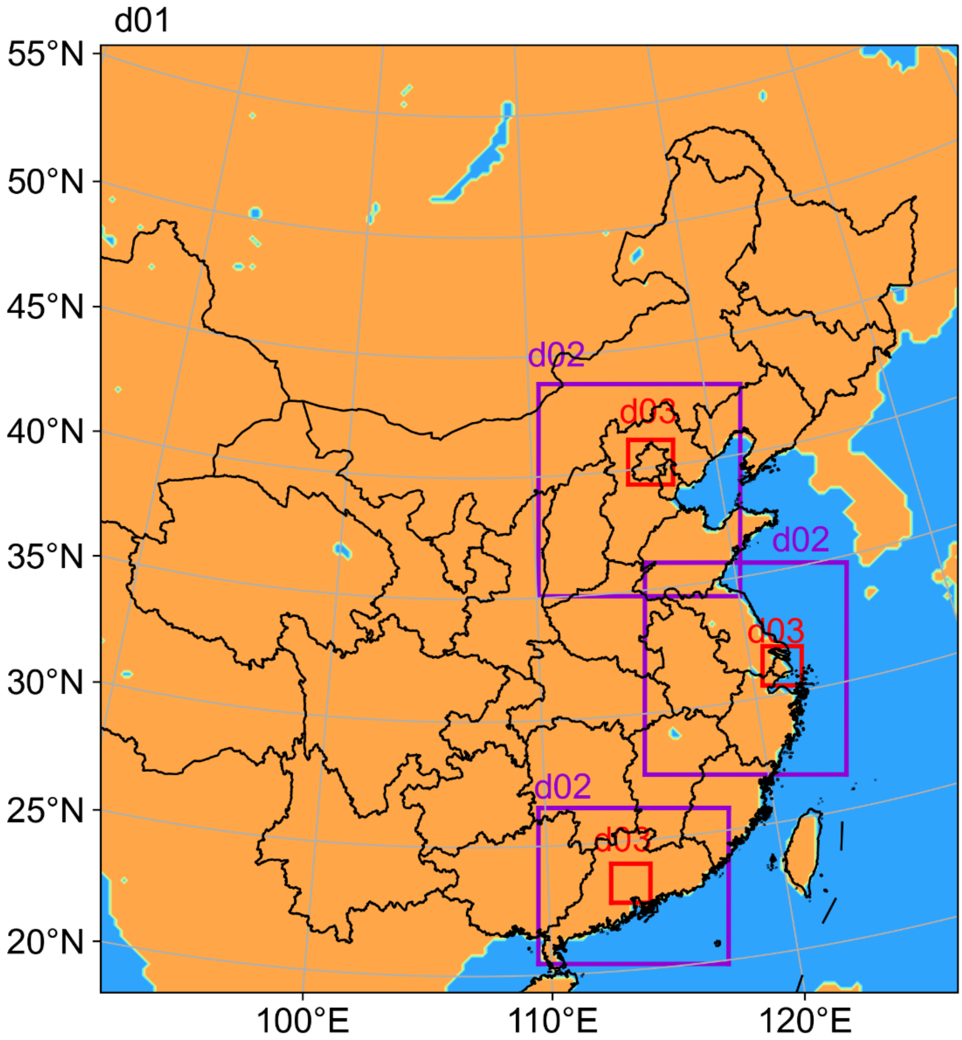

Figure S1. Simulation domains with grid spacings of 25 km (outer domain), 5 km (purple) and 1 km (red).

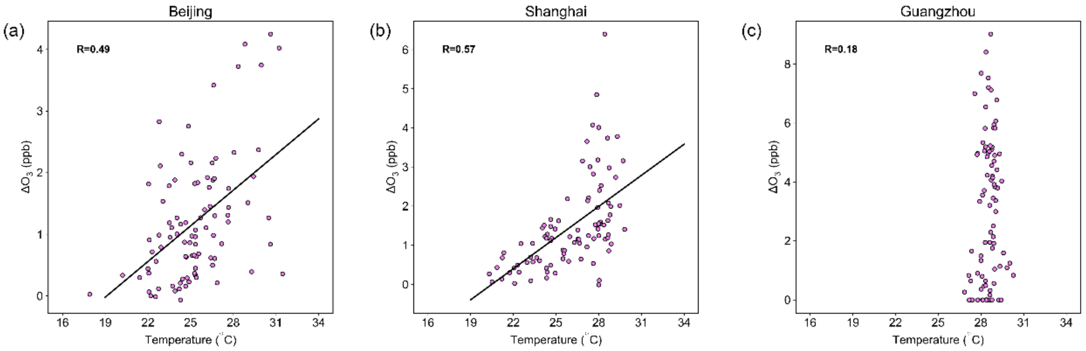

Figure S2. Relationship between 2 m temperature and ΔMDA8 O_3 in (a) Beijing, (b) Shanghai and (c) Guangzhou. Lines of best fit are plotted only where the correlation coefficients are significant (Beijing and Shanghai).

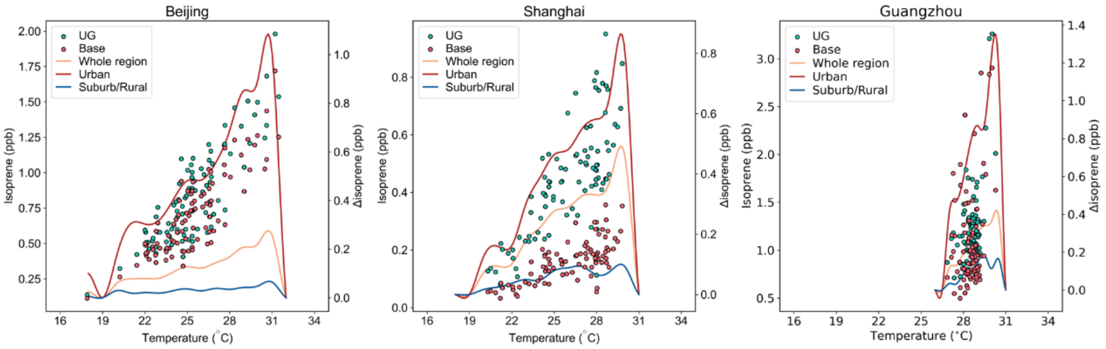

Figure S3. Relationship between 2 m temperature and isoprene in three cities. Here, the UG scenario takes into account emissions from urban greening while the Base scenario does not consider it.

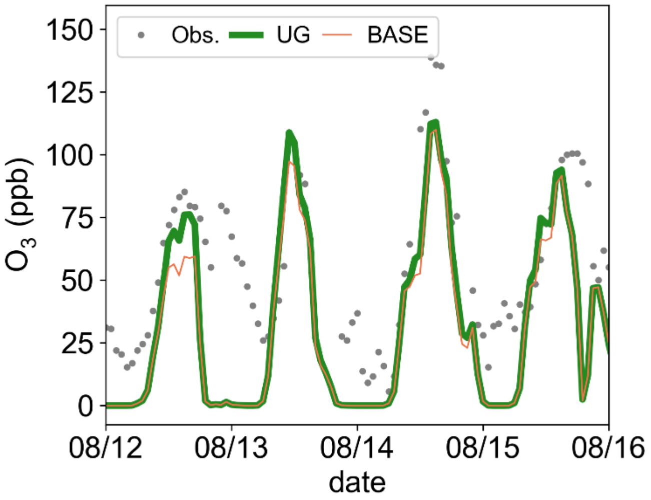

Figure S4. Time series of observational and simulated O_3 in Shanghai between 12 August and 15 August. Here, the UG scenario takes into account emissions from urban greening while the Base scenario does not consider it.

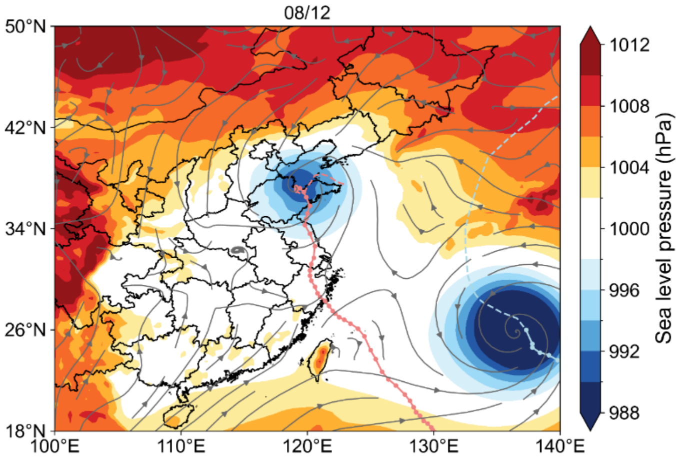

Figure S5. Averaged wind field and sea-level pressures on 12 August 2019. The pink line represent the track of typhoon "Lekima" while the light blue line represent the track of typhoon "Krosa".

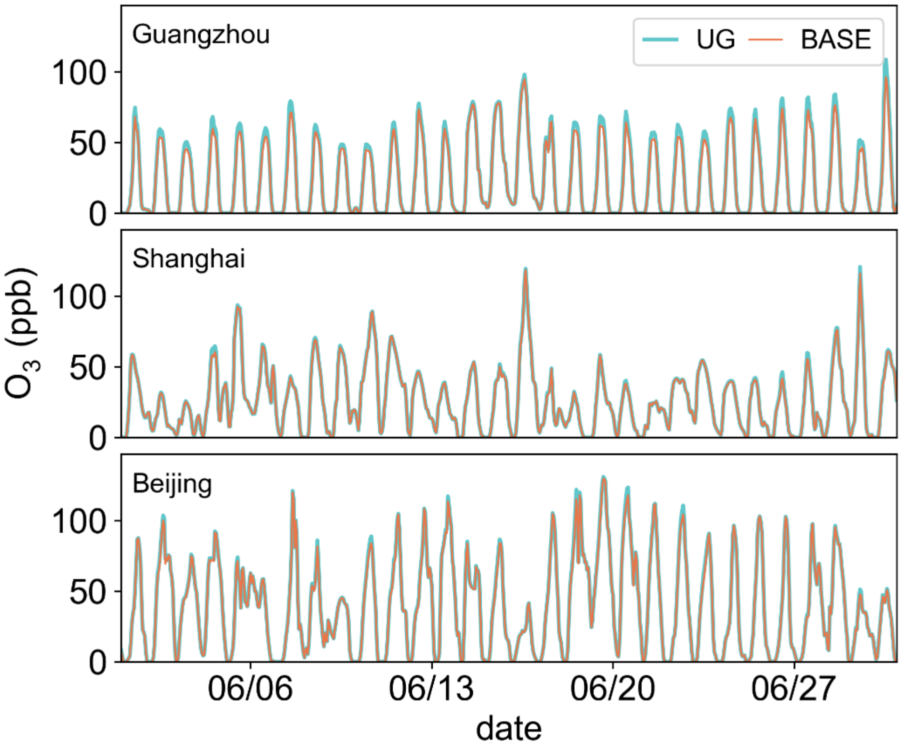

Figure S6. Time series of simulated O_3 in three cities in June. Here, the UG scenario takes into account emissions from urban greening while the BASE scenario does not consider it.
